# Development of Eco-Friendly Hydrophobic and Fouling-Release Coatings for Blue-Growth Environmental Applications: Synthesis, Mechanical Characterization and Biological Activity

**DOI:** 10.3390/gels8090528

**Published:** 2022-08-23

**Authors:** Silvia Sfameni, Giulia Rando, Alessia Marchetta, Cristina Scolaro, Simone Cappello, Clara Urzì, Annamaria Visco, Maria Rosaria Plutino

**Affiliations:** 1 Department of Engineering, University of Messina, Contrada di Dio, S. Agata, 98166 Messina, Italy; 2 Institute for the Study of Nanostructured Materials, ISMN—CNR, Palermo, c/o Department of ChiBioFarAm, University of Messina, Viale F. Stagno d’Alcontres 31, Vill. S. Agata, 98166 Messina, Italy; 3 Department of ChiBioFarAm, University of Messina, Viale F. Stagno d’Alcontres 31, Vill. S. Agata, 98166 Messina, Italy; 4 Institute for Biological Resource and Marine Biotechnology (IRBIM)-CNR of Messina, Spianata S. Raineri 86, 98122 Messina, Italy; 5 Institute for Polymers, Composites and Biomaterials-CNR IPCB, Via Paolo Gaifami 18, 95126 Catania, Italy

**Keywords:** sol-gel technique, fouling release, hydrophobicity, wettability, adhesion

## Abstract

The need to ensure adequate antifouling protection of the hull in the naval sector led to the development of real painting cycles, which involve the spreading of three layers of polymeric material on the hull surface exposed to the marine environment, specifically defined as primer, tie coat and final topcoat. It is already well known that coatings based on suitable silanes provide an efficient and non-toxic approach for the hydrophobic and antifouling/fouling release treatment of surfaces. In the present work, functional hydrophobic hybrid silica-based coatings (topcoats) were developed by using sol-gel technology and deposited on surfaces with the “doctor blade” method. In particular, those organic silanes, featuring opportune functional groups such as long (either fluorinated) alkyl chains, have a notable influence on surface wettability as showed in this study. Furthermore, the hydrophobic behavior of this functionalized coating was improved by introducing an intermediate commercial tie-coat layer between the primer and the topcoat, in order to decrease the wettability (i.e., decreasing the surface energy with a matching increase in the contact angle, CA) and to therefore make such coatings ideal for the design and development of fouling release paints. The hereby synthesized coatings were characterized by optical microscopy, contact angle analysis and a mechanical pull-off test to measure the adhesive power of the coating against a metal substrate typically used in the nautical sector. Analysis to evaluate the bacterial adhesion and the formation of microbial biofilm were related in laboratory and simulation (microcosm) scales, and assessed by SEM analysis.

## 1. Introduction

The undesired growth or deposition of marine (micro)organisms in all those surfaces and installations that operate in the marine environments (such as small or large boats, pylons, mining platforms, submarine monitoring systems, etc.), and which are subjected to the erosive action of atmospheric agents and chemical agents every day, is referred as “(bio)fouling” [1]. (Bio)fouling can be divided into two main and distinct categories, namely microfouling and macrofouling, according to the different types of marine organism involved. In particular, diatoms and bacteria are the primary causes of microfouling, whereas marine creatures (i.e., barnacles and tubeworms) and plants (algae) are rather responsible for macrofouling [2,3,4,5,6]. Algae and encrustations, defined as a whole as biofouling, causes serious problems to marine industries and exposed areas due to corrosion and hydrodynamic resistance, which leads to high fuel consumption and higher maintenance costs that should not be underestimated. Furthermore, all naval structures, port areas as well as submerged cultural heritage can suffer real damage over time due to Microbiologically Influenced Corrosion (MIC) by which bacteria modify and accelerate the kinetics of corrosive processes. Therefore, it is inevitable to reserve for these systems various biofouling defensive treatments, such as biocidal and non-biocidal paints that can shield the hull from algae and all varieties of marine life. The main and the most used strategy to inhibit marine fouling is to use Anti-Fouling (AF) paints containing biocides [7], i.e., a highly toxic substance that is incorporated into the ingredients of the paint and which in contact with sea water, due to chemical and physical phenomena, is released into the marine environment in a controlled way [8]. Even if the submerged surface of the hulls of all boats could be protected with potentially toxic antifouling paints, it must be considered the extent of this subtle and little-known source of environmental pollution. A remarkable example of an antifouling biocide that was regrettably still in use a few years ago, is tributyltin (TBT); nowadays it is defined jointly of the principal toxic substances introduced by man into the environment and which, recently, was finally banned by a regulation of the International Maritime Organization (IMO) which in 2008 sanctioned its disappearance from the paints of all boats. One of the first effects of the TBT ban was a significant increase in antifouling paints containing copper and other biocides (organic and organometallic) [9]. For years, coatings containing hexavalent chromium have also been used as anti-corrosion protection of surfaces, but increasingly stringent environmental standards in the industrialized world led to the abandonment of pre-treatments based on heavy metals and, in particular, those based on chromates due to the strong toxicity of hexavalent chromium. In recent years, however, research was always limited by the need to achieve a balance between the protection of these marine installations, operating close or under the sea, from the aggressiveness of the environment, and the simultaneous protection of the ecosystem from the pressures generated by human technologies. In light of this, business and environmental legislation are driving science and technology towards non-biocidal solutions based only on the physical-chemical and material properties of coatings [10,11,12]. Therefore, research aimed at developing antifouling eco-friendly and sustainable paints and coating, i.e., featuring lower environmental impact, was enhanced, but the truly environmentally friendly alternatives seem to be those showing Fouling Release (FR) activity, since they are able to prevent the settlement of biofouling organisms, without releasing any toxic substances, and thus preventing marine ecosystems and either protecting human health [13].

It was emphasized that in addition to the AF/FR qualities, these paints and coatings should also have noticeable mechanical characteristics that will enhance their adhesion to metallic or composite substrates once applied [14,15]. Unfortunately, formulations that have good release properties for marine organisms (FR paints) often fail to have good adhesion to the coated substrate as well. It is therefore important to design and develop a unique multicomponent material that manages to have both good adhesion and FR characteristics. In the naval sector, in fact, there is a technique for applying AF/FR paints and coatings on metallic hull surface, and it was also created specifically to address biofouling issues. The procedure consists in the application of three subsequent layers of polymeric material to the steel surface in contact with the sea, i.e., a primer, a tie-coat and a final top-coat. The final topcoat must have specific chemical-physical characteristics, in order to satisfy precise static and dynamic conditions, based on the principle of adhesion and resistance to abrasion by external stresses, including hydrodynamic effects that occur between the hull and the water. These three layers must be suitably bonded together with a prior preparation of the host surface, in such a way as to give longer life and higher strength [16].

**Figure 1:**
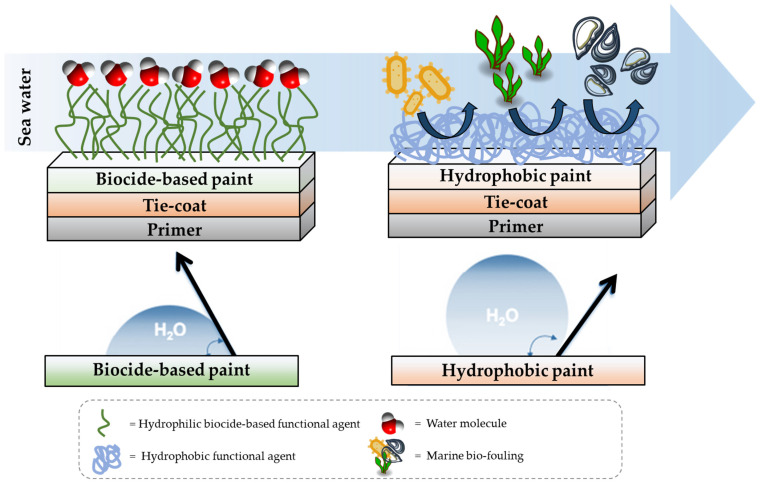
Anti-Fouling and Fouling Release activity of biocide-based and hydrophobic coatings, respectively.

In this regard, the approach to developing new coatings is to make a “deterrent” surface that inhibits the initial settlement phase of microorganisms, such as silicone coatings that produce an anti-stick effect, or either fluoropolymer coatings with non-stick effects or furthermore siloxane-based coatings that base their effectiveness essentially on low surface energy [17,18,19,20,21]. In recent years, numerous research studies [22,23,24,25,26] were conducted on the development of new fouling release (FR) technologies based on sol-gel technique for the synthesis of hydrophobic coatings containing fluorinated and alkyl alkoxysilanes featuring different lengths chains [27,28,29,30,31]. This fouling release technology is based on the concept of minimizing the adhesion force between the fouler and the material of which the hull surface is made, allowing the removal of the fouling simply through the motion of the ship during navigation [32,33,34,35]. Moreover, these FR alternative systems were widely used in biofouling mitigation treatment of different surface, providing a non-toxic alternative to biocidal-based AF coatings (see Figure 1).

In this paper, studies on the design, synthesis, deposition and mechanical and biological applications of sol-gel-based hybrid coatings with strong water-repellent properties were performed. The most important benefit of using the versatile sol-gel technique concerns the formation of a functional alkoxysilane layer (or xerogel) at room temperature [36,37,38,39,40,41,42].

In particular, the (3-Glycidyloxypropyl)trimethoxysilane (G) precursor [42,43,44,45,46] was employed as sol-gel cross-linker in combination with an equimolar amount of hydro repellent alkoxysilane, featuring different lengths of the hydrocarbon chain (namely, C16, C8, C2; see Table 1 reporting codes and molecular structures of each silane employed reagent), or a fluoro-containing alkoxylsilane (F3), in order to obtain the corresponding sol-gel G/C16, G/C8, G/C2, and G/F3, respectively. 

**Figure 2:**
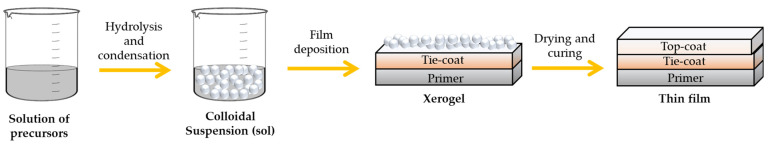
Subsequent stepwise sol-gel process for functional coating fabrication.

Therefore, to enhance the surface hydro repellency, the functional sol-gel was applied on the proper surface (i.e., glass slides or steel stub) as a top-coat, i.e., after the application of commercial primer and tie-coat (namely primer Jotacote Universal N10 and tie-coat Safeguard Universal ES, both supplied by Jotun Italia Srl), respectively, as shown in Figure 2. 

In detail, wettability and adhesion properties were investigated with the execution of two tests: the contact angle in distilled water and mechanical pull-off test. As a matter of fact, the application of the hybrid sol-gel based films obtained by using these alkyl (either fluorinated [47]) precursors, in combination with the selected commercial primer and tie-coat) allows us to obtain hydrophobicity values of the final top-coat higher than those obtainable by using previously available commercial antifouling paint, while also maintaining a fair resistance to external stress. At the same time, analysis to evaluate the bacterial adhesion and the formation of microbial biofilm on the developed coating were related at the laboratory (cultivation of marine bacteria) and simulation (microcosms) scale.

## 2. Results and Discussion

### 2.1. Sol-Gel Synthesis and Coating Deposition

**Figure 3:**
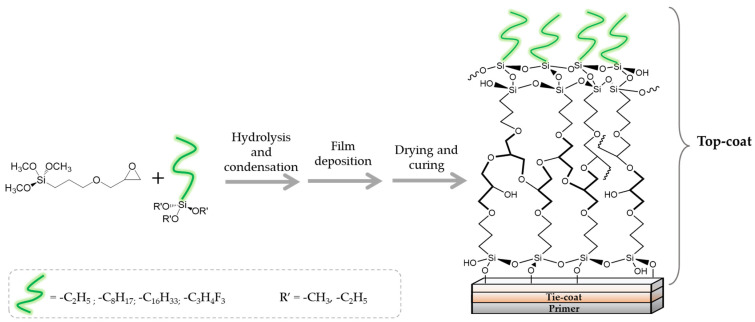
Sol-gel synthesis steps for hydrophobic coating preparation.

A conventional sol-gel synthesis was performed in order to develop the four designed functional hybrid silylated coatings. Thanks to its bifunctional behavior (i.e., related to the contemporary presence of an anchoring epoxy ring and a binding threemethoxysilyl group), the 3-glycidoxypropyltrimethoxysilane (G) precursor was employed and mixed with four alkoxysilanes (Table 1), differing for the length of the hydrocarbon chain or for the presence of fluoride atoms, with the aim to develop hydrophobic and fouling release coatings. The overall synthetic pathway toward the formation of hydrophobic alkoxysilane-based coatings is shown in Figure 3. GPTMS polymerization occurs with formation of a polyethylene oxide network (PEO), where the epoxy-ring opening reaction of the GPTMS molecules may be followed by subsequent steps of hydrolysis and condensation that bring to the subsequent alcoxysilane polymerization [48]. 

Through drying and curing processes, associated condensation reaction occurs as directly related with the removal of water from the polymer matrix, thus resulting in the formation of more stable silica-based 3D networks. 

**Figure 4:**
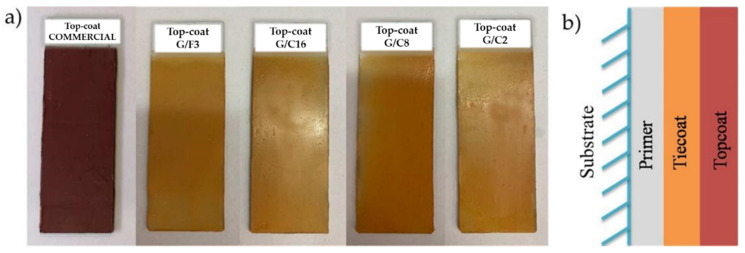
Images of different coating as deposited on the microscope slides (**a**); schematic summary of the individual layers (**b**).

The colloidal sol-gel solutions are finally used to coat either microscope slides or steel surfaces (see Figure 4).

In detail, the deposition phase includes three subsequent steps:

1. The application of a first layer of commercial primer Jotacote Universal N10 (supplied by Jotun Italia Srl), i.e., a two-component epoxy with polyamine hardener (3:1).
2. The application of an intermediate tie-coat (Safeguard Universal ES supplied by Jotun Italia Srl), i.e., a two-component epoxy vinyl with a polyamide hardener (5:1), which gives rise to a salmon pink color for the coated sample. The intermediate application of the tie coat serves to ensure adhesion between primer and top-coat.
3. The application of the developed silica-based hydrophobic top-coat (commercial antifouling or G/F3- G/C16- G/C8- G/C2 coatings). The commercial antifouling or fouling release topcoat chosen for this work is the Sea Quantum Ultra S, single component with chemical reaction of silylacrylate, supplied by Jotun Italia Srl.

**Figure 5:**
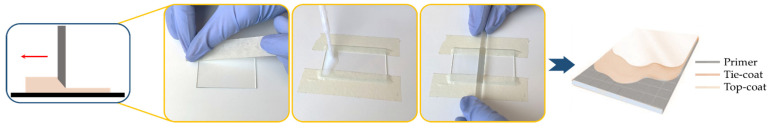
Doctor blade method for functional coating deposition.

Figure 5 shows the microscope slides covered with the sol-gel G/F3, G/C16, G/C8, G/C2 coatings together with the commercial top-coat set on the left side, as carefully prepared for the experimentation (a), together with a scheme of the individual layers succession (b).

To ensure greater protection, two layers of each component were deposited, adequately respecting the drying times of the layers. After the application of primer and tie-coat, a milliliter of each of the sol-gel matrices was roller-coated (doctor blade method) as the third and last layer on each pre-treated slide (Figure 5) and then allowed to cure at room temperature for 24 h or at T = 105 °C for 30′ following by further 30′ at T = 180 °C. 

### 2.2. Structural Characterization

**Figure 6:**
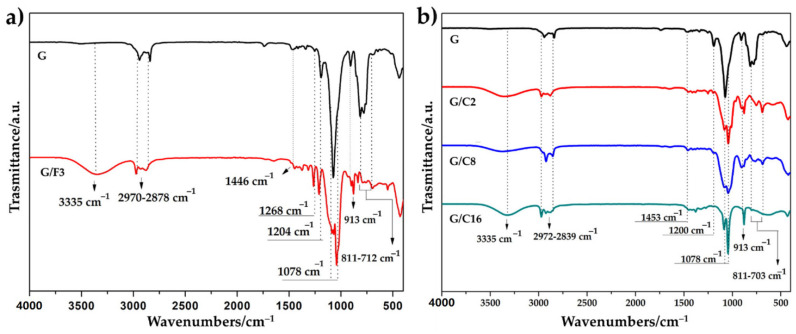
FT-IR spectra of glass slides treated with G and G/F3 sol (**a**) and G/C2, G/C8 and G/C16 sol (**b**).

The chemical structure of the applied coatings was investigated by ATR FT-IR analysis and the results are shown in Figure 6. 

In the coating realized by adding a fluorinated precursor, such as trifluorobutyl-trimethoxysilane (F3) to the 3-Glycidyl-oxypropyl-trimethoxysilane (G), the occurrence of fluorine was confirmed by the variation in spectra in the range of 1100–1270 cm^−1^, owing to the presence of the peak at 1268 cm^−1^ attributed to the carbon-fluorine stretching modes (C-F). 

Compared with the FT-IR spectra of (3-Glycidyloxypropyl)trimethoxysilane (G), FT-IR spectrum of G/F3, G/C16, G/C8 and G/C2 shows also the appearance of transmission of some characteristic bands at 1078 cm^−1^, 857–760 cm^−1^, 811–703 cm^−1^ corresponding to the siloxane stretching and bending modes (Si-O-Si), confirming the formation of an inorganic siloxane matrix and the peak at 913 cm^−1^ (Si-O-H). Frequency at 3335 cm^−1^ denoted the existence of O-H groups of Si-OH from the hydrolysis reaction. The sharp peaks at 2972 cm^−1^ and around 2839 cm^−1^ are due to C-H symmetric stretching asymmetric stretching vibrations.

### 2.3. Surface Characterization

**Figure 7:**
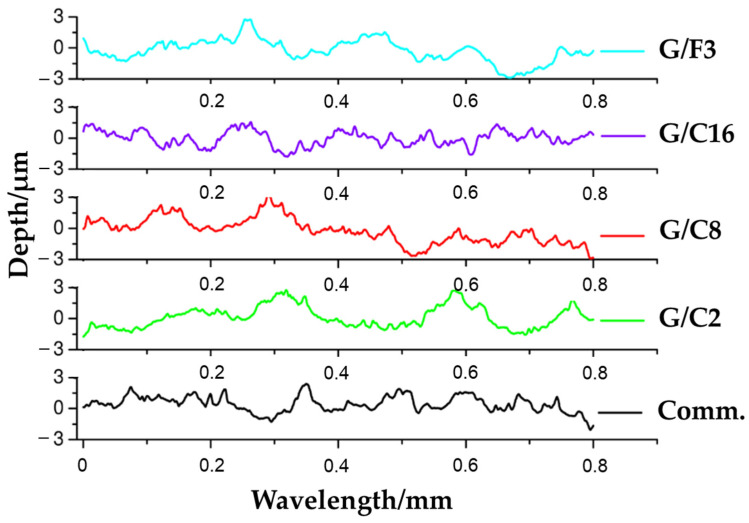
Roughness profiles of antifouling coatings.

In order to fully understand the chemical structure influence of the coating, the wettability and surface roughness need to be measured at the same sample spot and the results, evaluated according to Equation (2) as reported in Materials and Methods, to separate the effect of the roughness to the wettability. In Figure 7, the surface profile of the antifouling coatings is shown, as measured with a surface profilometer. The roughness of samples prepared by bar deposition depends not only on the composition of the coatings but also on the deposition parameters. 

The surface roughness parameters of these coatings (Ra) are listed in Table 2.

It is well-known that roughness affects the wettability because it amplifies the wetting effect of the surface chemistry, making the evaluation of the coating complex [49]. The statistical analysis showed that the determined values of the contact angle and roughness for all the samples are highly significant (*p* < 0.0001).

The sample G/C16 is the least rough sample (Ra = 0.53 μm, *p* < 0.0001) compared to all the other coatings whose roughness ranges between 0.73 μm (Comm., *p* < 0.0001) and 0.96 μm (G/C8, *p* < 0.0001).

Table 2 shows the contact angle (θ) values given by Wenzel’s equation and Young’s equation concerning the commercial topcoat and the sol-gel coatings. 

**Figure 8:**
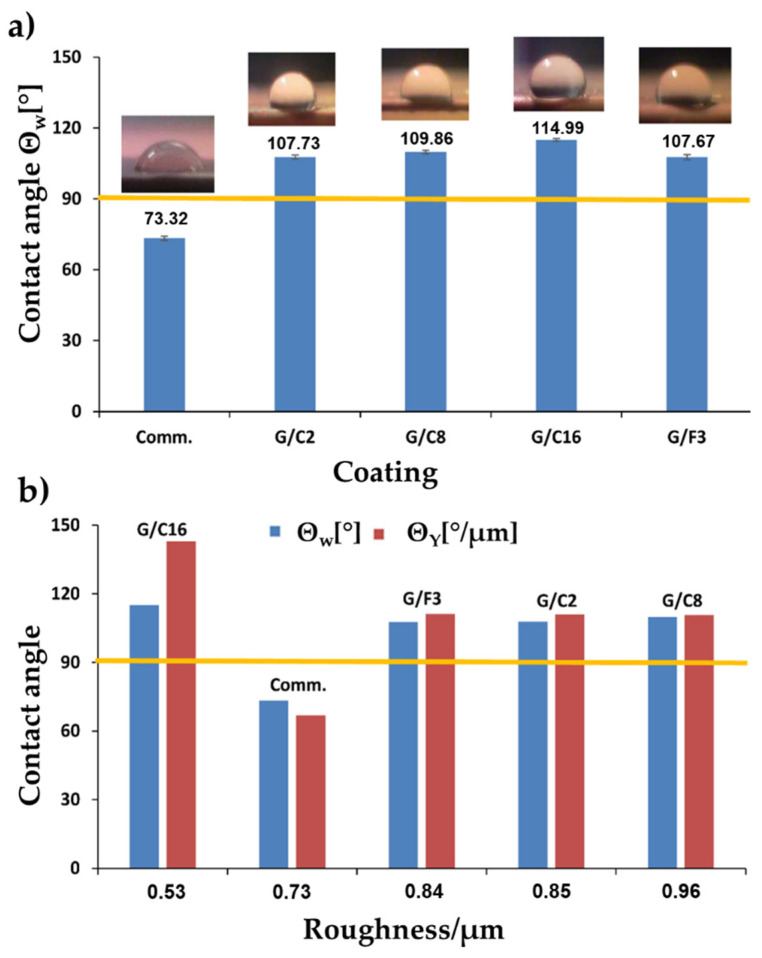
Histogram of the contact angle θ_W_ of coatings with photos of the representative drops (**a**); comparison of contact angles θ_W_ and θ_Y_ vs roughness of coatings (**b**).

Inversely, the commercial coating has a hydrophilic behavior since θ_w_ = 73.32° (*p* < 0.0001). All the other developed coatings instead exhibit a hydrophobic behavior, being their contact angle higher than 90°. In particular, the most hydrophobic one is the G/C16 sample whose θ_W_ = 114.99° (*p* < 0.0001) (Figure 8).

The Wenzel contact angle values of the G/C2, G/C8, G/F3 coatings are very close to each other (θ_W_ ≅ 108 ± 2°). All the sol-gel coatings show an increase in the contact angle (Δ% = 31.90% for G/F3 − Δ% = 49.83% for G/C8), and of the consequent hydrophobicity. In particular, the G/C16 substrate exhibit a greater increase in the θ_Y_ (114.99°, Δ% = 56.83%, *p* < 0.0001) with respect to that θ_w_ (142.85°). The ideal contact angle value of each sample, represented by the Young value (θ_Y_) is always higher than the Wenzel’s one (θ_w_), see Figure 8 and Table 2. This because it is referred to an ideal “perfectly-smoothed” surface.

As it can be seen from all the above discussed results, with the increase in the length of the long hydrocarbon chain of the alkyl-silanes, a significant increase in the contact angle of the treated substrates is observed. Thus, the hydrocarbon chain length can be directly related to the hydrophobic behavior of the coated samples, by following the same trend:

C16 > C8 > C2

**Figure 9:**
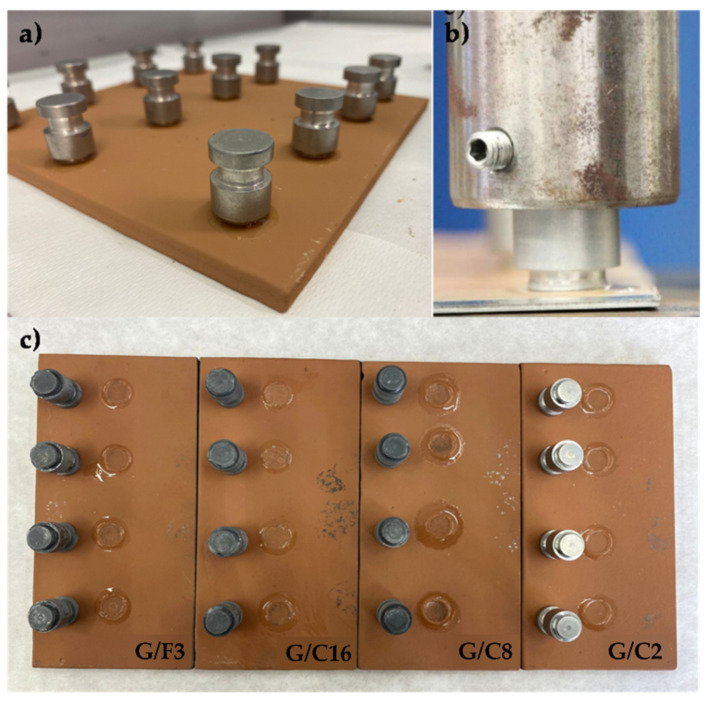
Pull-off test of the G/F3 coating (**a**); fixing phase of the dolly in the dynamometer (**b**); image of the dolly detached after the pull off test (**c**).

Pull-off adhesion testing was used to assess the mechanical performances of the coatings. Low adhesion values are indicative of premature failure of the coating. The pull-off method for adhesion testing involves, initially, gluing a test dolly to the coated deposited in a small circular area of 1.5 mm diameter, on the Dh36 steel metal specimens, previously fully coated with primer and tie-coat (Figure 9a). Thereafter, the dolly, attached to a mating connector of the dynamometer (Figure 9b), is pulled by a force perpendicular to the surface. The force at which this occurs the removal of the dolly from the substrate and the type of failure obtained is recorded using a stress/strain curve as a measure of the adhesion properties of the coating.

The nature and the type of fracture of the dolly from the substrate are evaluated, through visual inspection and according to normative ASTM D4541-02 or ISO4624:2016 (Figure 9c).

**Figure 10:**
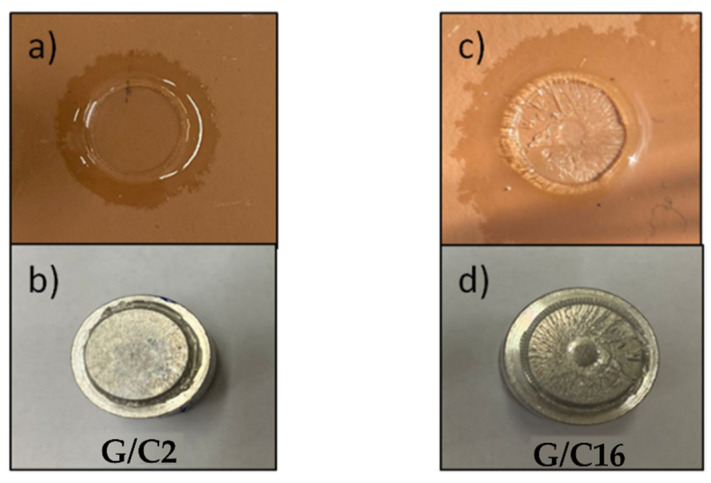
Substrates images and dollies after the pull off test, with adhesive properties sample (**a**,**b**) and cohesive properties (**c**,**d**) of G/C2 and G/C16 sample, respectively.

The examination of each dolly and the relative adhesion area showed a usual adhesive break at the support/coating interface for the samples G/C16, G/C8. Instead, the sample G/C2 and G/F3 showed a break at inside the lining, suggesting a cohesive break. The evaluation of the images suggested us that the samples who gives an adhesive break exhibit the best mechanical adhesion (Figure 10).

**Figure 11:**
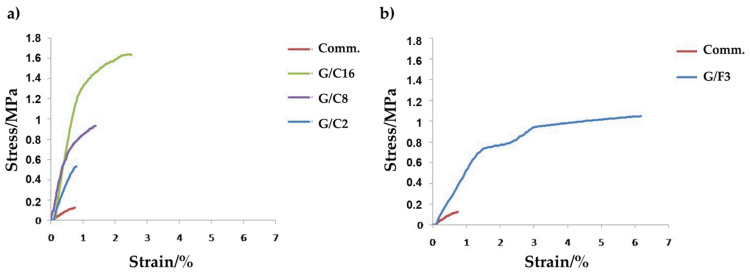
Stress/strain curves of the coatings G/C16, G/C8 and G/C2 (**a**) and G/F3 (**b**), compared to commercial coating.

Based on the results of the pull-off test, a graphical comparison of the adhesive properties of the coatings G/C16, G/C8, G/C2, G/F3 and the commercial finish can be made (Figure 11). Statistical analysis confirmed that the mechanical parameters of the pull off test of the coatings are highly significant for all samples (*p* < 0.0001).

Table 3 shows the main mechanical parameters, indicated in detail in the Material and Methods section.

The stress-strain curves of the coatings (G/C2, G/C8, G/C16 and G/F3) all show better adhesion than the commercial coating. In detail, commercial paint has a modest percentage elongation (ε_r_% = 10.43, *p* < 0.0001) and reaches the yield point with a stress of only 0.13 MPa (*p* < 0.0001), and therefore plastic deformation.

On the other hand, all the coatings we synthesize have a good percentage of deformation at break, between 16% and 32% (*p* < 0.0001). Coatings G/F3 and G/C16 have a particularly high tensile strength, reaching a value of 0.61 MPa and 1.20 MPa (*p* < 0.0001), respectively. The yield values are greater than the commercial coating; therefore, it increases their resilience, thus demonstrating a good compromise between stress and deformation. On the other hand, sample G/C2 shows little significant tensile strength (0.47 MPa) due to poor adhesion of the coating to the substrate, according to the images of Figure 11a,b.

In general, therefore, the synthetized coatings have a ductile character, which allows them to obtain a specific degree of deformation as a function of the applied tensile stress. The best adhesion feature was reached in the G/C16 sample.

### 2.4. Bacterial Tests

#### 2.4.1. Contact and Environmental Toxicity of the New Functionalized Coatings

**Figure 12:**
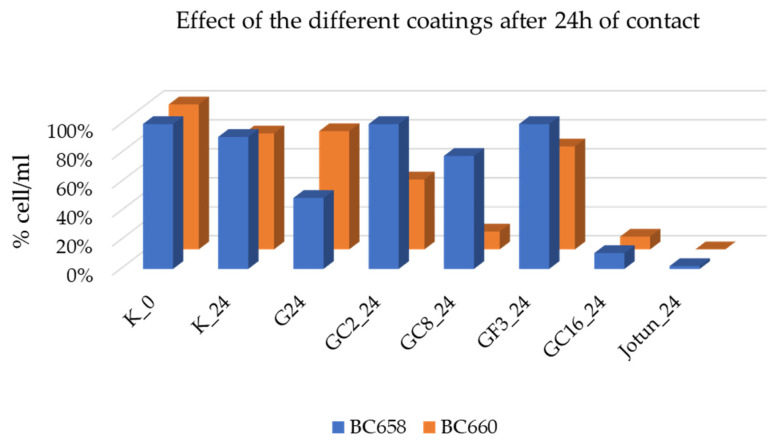
Bacterial strains behavior after 24 h of contact with the untreated (K) and treated with functionalized coatings glass slides. Bacteria are: a Gram—strain *Stenotrophomonas maltophilia* BC 658 and Gram + strain *Rossellomorea aquimaris* BC 660. Strains growth was expressed in percentage of viable and culturable cell/mL.

**Figure 13:**
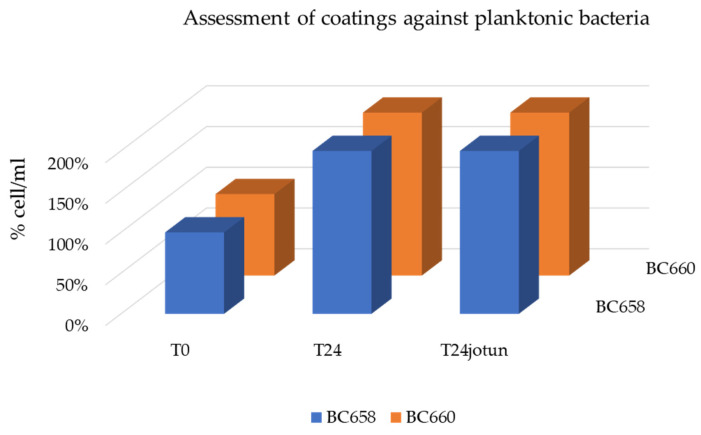
Planktonic bacterial growth after 24 h of incubation in presence of treated glass slides. No toxicity effects due to the release of the coatings in the aquatic environments was observed after 24 h of incubation in continuous agitation at 30 °C. Experiments with the commercial coating Jotun were carried separately.

Results obtained during the short-term laboratory tests are shown in Figure 12 and Figure 13.

In Figure 12, the results, expressed as percentage of viable and culturable cells (cfu/mL), show that for the untreated controls the rate of survival cells is 91% after 24 h of contact, while the treated surfaces show a different behavior depending both from the type of bacterial strains and of the coatings tested. In particular, coatings G/C16 and the commercial one Jotun showed a remarkable reduction in the survival bacteria (9 and 11% for Gram + and Gram−, respectively) for the G/C16 and 100% of death for the commercial coating Jotun against both type of bacteria.

Regarding the eventual toxicity of coatings released in the liquid environment that could affect the planktonic cells, as shown in Figure 13, no toxicity, expressed as percentage of viable cell/mL, was observed as demonstrated by the 10-fold growth of bacteria in the system after 24 h of incubation in presence of all treatments including the commercial one Jotun as compared to the initial number of cells/mL (=100%). 

**Figure 14:**
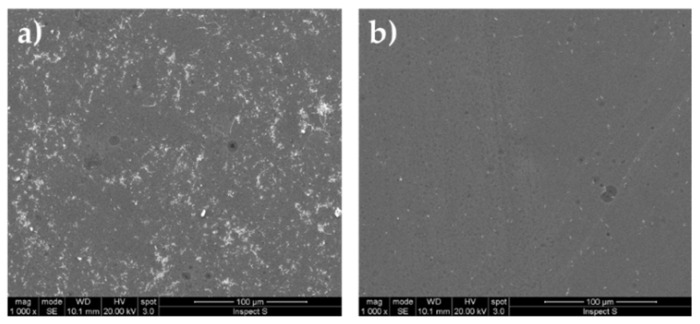
Controls. Adhesion on untreated glasses of bacteria: (**a**) BC 658 (Gram−) (**b**) BC 660 (Gram+).

**Figure 15:**
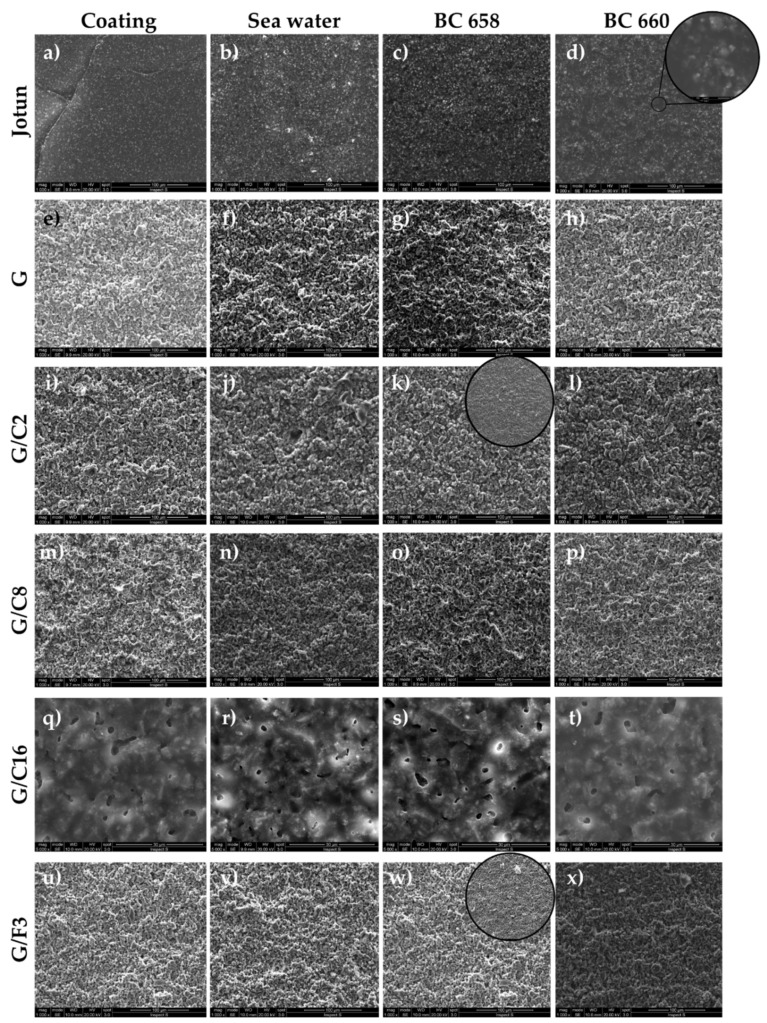
Adhesion of bacteria on treated glasses with coatings (commercial Jotun, (**a**–**d**); G, (**e**–**h**); G/C2, (**i**–**l**); G/C8, (**m**–**p**); G/C16, (**q**–**t**); G/F3, (**u**–**x**)). The first column shows the coatings after deposition; the 2nd column shows the coatings after immerging for 24 h in sterile sea water; the 3rd column are the images of the behavior of coating surfaces against the adhesion of strain BC 658 (Gram−); the 4th column in presence of a suspension of the strain BC 660 (Gram+). The circular images at higher magnification (30 μm) the bacteria adhering on the surfaces.

Regarding the occurrence of adhering cells within the 24 h of immersion, considered as first step for the biofilm production and microfouling activity, results are shown in Figure 14 and Figure 15.

In the untreated control, the Gram—strain, as it was observed under SEM, formed a heavy biofilm at the interface between liquid and air (Figure 14a) while a scarce adhesion was observed in the fully submerged part of the untreated slide. No relevant biofilm formation was observed in the untreated control for the Gram + strain, but sparse isolated cells (Figure 14b). 

The observations under SEM of the treated slide glasses with different coating showed that within 24 h of incubation only a scarce number of cells were able to adhere to the different surfaces (Figure 15) if compared with the adhesion occurring on untreated glass surfaces (Figure 14). Bacteria were clearly seen at higher magnification (30 μm) as shown for the commercial coating and strain BC 660 (Figure 15d), coating G/C2 in presence of strain BC 658 (Figure 15k) and G/F3 and strain BC 658 (Figure 15w). 

It is interesting to note that for the coating G/C16, the cells of the Gram—strain BC658 were seen allocated within the pores of the coating (Figure 15s). In particular, the G/C16 coating is characterized by a different surface morphology than the others developed in this study, thus showing a distinguish surface featuring different pores that lead to the entrapping of bacterial cells. Currently, other studies are in progress to further understand clearly the mechanism of adhesion onto this coating.

#### 2.4.2. Medium Term Microbial Adhesion in the Microcosm

**Figure 16:**
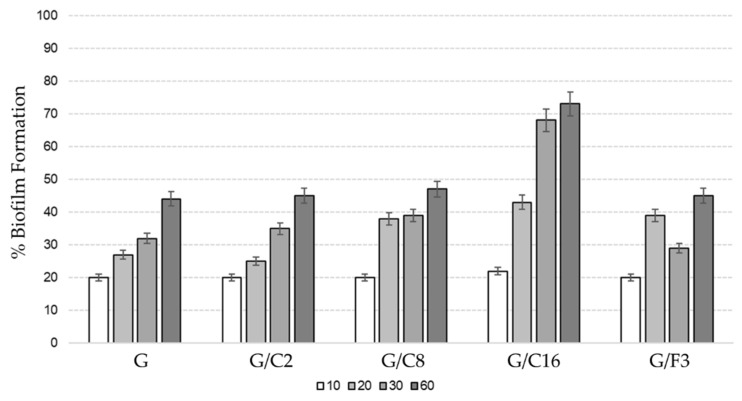
Percentage of microbial biofilm formation observed (with direct DAPI count) on the surfaces analyzed in this study. Different bars color identified different experimental time (10, 20, 30 and 60 days).

**Figure 17:**
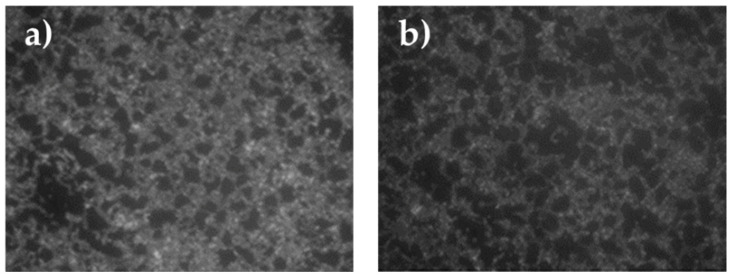
Microbial biofilm formation observed (with direct DAPI count) on the surfaces analyzed in this study (100× immersion) after 60 days of immersion. (**a**) G/C16, (**b**) G/F3.

The regular observation of glass slides submerged in the tank of the microcosm systems evidence as the different composition of coatings influence the biofilm formation (Figure 16 and Figure 17) along the experimental time up to 60 days. In general, all coatings except G/C16 show a dynamic similar to that of control (G); only samples treated with coating G/C16 showed an important increment of the adhesion rate up to ~70%, T_60_). 

## 3. Conclusions

This work aimed to develop a new antifouling system that uses the sol-gel method for the synthesis of new finishes based on fluorinated and long alkyl chain alkoxysilanes with chains of various lengths. These types of formulations are widely used for their hydrophobic actions. In particular, in the first phase of this work, we tested a simple process for the production of hybrid sol-gel coatings, which were applied as topcoats on substrates pretreated with primer and tie-coat, showing improved performance compared to the commercial one. Moreover, in this study it is found that suitable functional groups in organic silanes such as long-chain alkylsilanes have a considerable influence on surface wettability. 

The application of hybrid films obtained by adding long-chain alkylsilanes allows us to obtain hydrophobicity values comparable to those obtainable with traditional fluorinated precursors, as reported in a previous work [47], while maintaining a fair resistance to external stress. Furthermore, the hydrophobic behavior of these coated products is improved by introducing an intermediate and commercial tie-coat layer between the primer first layer (close to the substrate to be coated) and the developed topcoats, in order to further increase the contact angle, while decreasing surface energy and wettability, and therefore making these eco-friendly coatings ideal for development of fouling-release paints/coatings. It is important to note that none of the functional silane-based developed and tested coatings release toxic compounds into the environment, even if this result will be further investigated and, in any case, it will be evaluated in a long-term experimentation.

## 4. Materials and Methods

### 4.1. Materials and Sol-Gel Synthesis

The (3-Glycidyloxypropyl)trimethoxysilane(G), (3,3,3-trifluoro-propyl) trimethoxy silane (F3), Hexadecyltrimethoxysilane (C16), Triethoxy(octyl)silane (C8) and Triethoxy(ethyl)silane (C2), were all purchased at the highest purity level and used as received by Sigma Aldrich, without any further purification. Commercial primer Jotacote Universal N10 and tie-coat Safeguard Universal ES were supplied by Jotun Italia Srl. 

A typical procedure [44,45,46] to prepare the sol-gel solutions is as follows: the (3-Glycidyloxypropyl)trimethoxysilane (G) precursor undergoes hydrolysis–condensation reactions in several steps in combination with an equimolar amount of a fluoro-alkylsilane (F3) or three different alkoxysilanes, which differ according to the length of the hydrocarbon chain (namely, C16, C8, C2), yielding the corresponding samples G/F3, G/C16, G/C8 and G/C2, respectively. Ethanol 96% vol. from Sigma Aldrich and deionized water were used as dilution media while HCl 37% was added dropwise to induce the hydrolysis-condensation reaction. 

The resulting mixture was vigorously stirred at room temperature for 12 h, so ultra-sonicated for 30 min to induce a homogeneous suspension.

Microscope slides (thickness 1 mm; size 76 mm × 26 mm) were pretreated with a piranha solution able to clean the surface from any type of organic and fat residue and, at the same time, to make the glass hydrophilic by hydroxylation of the surface and then washed with distilled water and left to dry in an oven for a few hours before the coating were applied.

### 4.2. Characterization and Testing

*ATR FT-IR analysis:* Fourier transform infrared analysis was performed to determine the chemical structure of the coatings. FT-IR spectra were acquired by using a V-6600 Jasco Spectrometer, equipped with an attenuated Total Reflection (ATR) accessory and they were recorded, at room temperature, in the spectra range of 4000–500 cm^−1^. 

*Wettability:* The wettability of the sol-gel coating on the microscope slides was evaluated by measuring ten times the height h (mm) and the base diameter d (mm) of 1 μL drop of deionized water on the horizontal surface of the sample, by means of a microlithic syringe (Hamilton, 10 μL). Wenzel, θ_W_ and Young, θ_Y_, contact angles were evaluated by the sessile drop method (ASTM D7334) [50,51,52]:

(1) $${\;\theta }_{w}=2\mathrm{arctg}\left(\frac{2h}{d}\right)$$

(2) $$\theta_{Y}=\mathrm{arcos}\left(\frac{{\cos\theta }_{w}}{r}\right)$$

where r is the surface roughness.

*Adhesion test:* The adhesion test was performed by a Lloyd LR10K universal testing machine (trademark of AMETEK Test & Calibration Instruments), according to ASTM D4541 by attaching steel metal dolls, perpendicularly on DH36 steel metal samples (fully coated with a commercial primer and tie-coat), to a topcoat layer to be tested. The following experimental conditions were used: Load cell 10 KN, Pre-load 1.00 N, Speed 1 mm/min, Diameter ≈ 9.88 mm, Breakage: load yields up to 20%. Three samples (for each type) of at least six dollies were extracted, by gaining specific mechanical parameters: E is the Young’s modulus [MPa]; σ_max_ is the maximum stress [MPa] σ_r_ is the stress at break [MPa]; ε_r_% is the percentage deformation at break [%]; *load r* is the load at break [N]; Wr is the work at break [J]. The statistical analysis showed that the mechanical parameters determined by the coatings pull off test, for all the samples are highly significant (*p* < 0.0001).

*Surface Roughness:* The surface roughness (Ra) was calculated by the portable and compact roughness tester, Surftest SJ-210- Series 178:

(3) $$\mathrm{Ra}=\frac{1}{N}\overset{n}{\underset{i=1}{\sum }}\left|\mathrm{Yi}\right|$$

where Ra is the arithmetic means of the absolute values of the deviations of the evaluation profile (Yi) from the mean line. The measurement conditions of the instrument were set according to the JIS2001 roughness standard: the roughness R profile for compliance, λs = 2.5 μm, λc = 0.8 μm, five sampling lengths and a stylus translation speed of 0.5 mm/sec. On average, n. 10 roughness profiles per type of sample were performed and then an average profile was obtained.

The mean differences and standard deviations of wettability, roughness and adhesion test were calculated. Data were first verified with the D’Agostino & Pearson test for the normality of the distribution and the Levene test for the homogeneity of variances. Data were normally distributed and homogenous; therefore, they were statistically analyzed by using one-way analysis of variance (ANOVA) and Bonferroni post hoc test for multiple comparisons at a level of significance set at *p* < 0.05 (Prism 8.4.1; GraphPad Software, Inc, La Jolla, CA, USA).

### 4.3. Assessment of Biocidal and Antifouling Activity

In order to test the characteristics of the new coatings against microfoulers, different tests were made as it is described below. 

#### 4.3.1. Short Term 24 h Assessment of Biocidal and Antifouling Activity

*Microbial strains*: Two bacterial strains *Stenotrophomonas maltophilia* BC658 and *Rossellomorea aquimaris* BC660 (Genbank accession numbers KY610289 and KY610290, respectively) were used in this study. Both strains were isolated from marine environment and maintained in the collection of the Department of CHIBIOFARAM of University of Messina (Italy). 

*Microbial suspensions*: Fresh bacterial suspensions of *Stenotrophomonas maltophilia* and *Rossellomorea aquimaris* were prepared after growing in Marine Agar medium (Condalab, Madrid, Spain) for 24–48 h at 30 °C. Bacterial colonies were then suspended in phosphate buffer solution (PBS, 0.01 M and pH 7.0), pellet was collected by centrifugation in Beckmann centrifuge at 10,000 rpm per 10′ at 4 °C and rinsed 3 times by repeating this step. Pellet was suspended in PBS in order to reach an OD_550_ of 0.125 corresponding to 1.5 × 10^8^ cfu/mL of 0.5 and OD_550_ of 1.5 corresponding to 1.8 × 10^9^ cfu/mL, respectively. The freshly prepared bacterial suspensions were then used for the following short-term test.

*Evaluation of biocidal activity of the new functionalized coatings*: Biocidal activity of the coatings was tested via the contact toxicity test carried out as described by Pistone et al. [18]. Untreated and treated glass slides with the different coatings G/F3, G/C2, G/C8, G/C16 were used; further slides with only G and slides with an already available commercial coating were used as comparative controls. Treated and untreated slides were then sterilized under UV light for 2 h. Subsequently, 200 µL of bacterial suspensions adjusted at the OD_550_ of 0.125 in PBS solution was placed on the surfaces of each untreated and treated slides. Each test was conducted in duplicate.

The specimens were placed in sterile glass Petri dishes containing a paper disc moistened with sterile water to avoid drops drying, and incubated for 24 h at room temperature (~25 °C).

After 24 h of contact, to evaluate the survival of bacterial strains, 10 µL were taken from each suspension and decimal dilutions were conducted in physiological solution + Tween 80 0.001%. A volume of 10 µL of each dilution was inoculated in duplicate in TSA (Tryptone Soy Agar, Codalab, Spain) plates and then incubated for 24–48 h at 30 °C. After incubation, colonies were counted to determine the number of viable bacterial cells (CFU/mL) after 24 h of contact. The eventual bactericidal effect was determined as percentage of difference between the CFU/mL obtained for each coating and the CFU/mL of negative control at T_0_ and T_24_.

*Assessment of Antifouling Activity and Toxic effect on planktonic bacteria*: To test the antifouling properties of the newly synthesized coatings, the untreated and treated glass slides sterilized under UV as above described, were placed inclined (30° angle) in a sterile glass container; 2 L of sterile Marine Broth (Condalab, Spain) was poured into the container. The experiments were conducted in parallel with each bacterial strain by inoculating 2 mL of each fresh bacterial suspension (1.5 OD_550_) to obtain a final concentration of 1.5 × 10^7^ cfu/mL. All the experiments were carried out in double. Incubation was carried in continuous slow agitation at 30 °C for 24 h.

After incubation, the slides were removed, rinsed with distilled water and prepared for the Scanning Electron Microscope (SEM) carried out as described by Plutino et al. (under submission). Briefly, slides were fixed overnight with 2.5% glutaraldehyde in 0.01 M phosphate buffer, then washed with 0.1 M phosphate buffer pH 7.0 and dehydrated by successive steps in increasing series of ethanol solutions: 30%, 50%, 70% and absolute and then air dried. 

*SEM analysis:* Prior to analysis, the samples were coated with chromium using a sputter coater. SEM measurements were acquired with a FEI Inspect S instrument coupled with an Oxford INCA PentaFETx3 EDX spectrometer, with a resolution of 137 eV at 5.9 keV (Mn Kα1) and equipped with a nitrogen cooled Si (Li) detector. The spectral data were acquired at a working distance of 10 mm with an acceleration voltage of 20 kV, counting times of 60 s, with approximately 3000 counts per second. The results were processed by the INCA energy software.

The antibiotic activity of coatings against planktonic bacterial cells was evaluated by inoculating serial dilutions of bacterial suspensions at the beginning of the experiments (T = 0) and after 24 h (T = 24 h) in TSA plates and the number of vital cells before and at the end of the experimental set was compared. 

#### 4.3.2. Medium-Term Bacterial Adhesion Tests in a Microcosm

**Figure 18:**
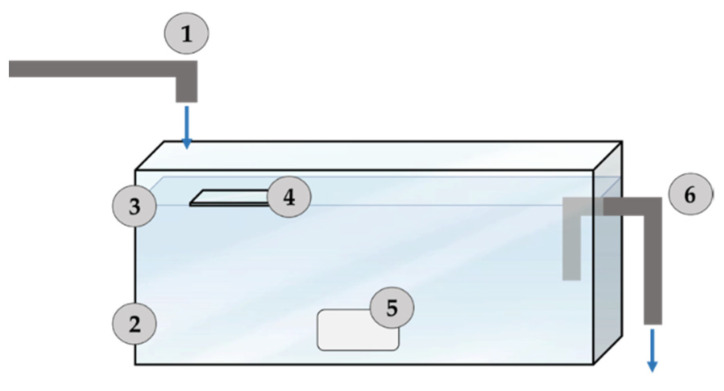
Hydraulic-engineering scheme of the experimental microcosms in use. (1) Water loading system (5 Lh^−1^); (2) Experimentation tank; (3) Experimental water level (90 L); (4) study slides; (5) internal recirculation pump (5 Lh^−1^); (6) “Overflow” system for water drainage.

All the experiments were carried out in Messina (Italy) at the “Mesocosm Facility” of IRBIM-CNR of Messina. The experiments were performed in rectangular glass tanks of 90 L capacity (100 cm long, 30 cm deep, 30 cm wide) (Figure 18).

Each microcosm was filled with 70 L of natural seawater collected directly by a pipeline from the harbor of Messina, Italy. The natural seawater was filled in continuous (5 Lh^−1^) for all experimentation time; the natural seawater was filtered through a 200 μm nylon mesh to remove large metazoans and detritus. The average water temperature was 18.5–19.5 °C, with daily temperature fluctuations of water not exceeding 2 °C. Microcosms were lighted by a fluorescent lamp, which consisted of six tubes (36 W, 120 cm) providing light on a 12/12 h of light/dark period. Microcosm water was mixed by a pump (35 Lh^−1^), placed at the exit of each tank, that takes water from two opposite bottom corners and drives it below the surface. 

Slides covered with the sol-gel G/F3, G/C16, G/C8, G/C2 coatings together with the commercial topcoat were insert inside the experimental tanks (Figure 18). After 10, 20, 30 and 60 days of experimentation slides were collected and analyzed in optical fluorescence microscopy for evaluate the biofilm formation. 

Slides were fixed with formaldehyde (2% final concentration), according to Porter and Feig (1980) and then stained with DAPI (4′,6-diamidino-2-phenylindole 2HCl, Sigma-Aldrich, Milan, Italy) and examined by epifluorescence with an Axioplan 2 Imaging (Zeiss) microscope (Carl Zeiss, Thornwood, NY, USA). Results were expressed as percentage of the number of cells present in the different area examined.

## Figures and Tables

**Table 1:** Sol-gel alkoxysilane precursors.

Name	Code	Molecular Structure
(3-Glycidyloxypropyl) trimethoxysilane	G	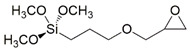
Triethoxy(ethyl)silane	C2	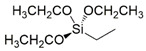
Triethoxy(octyl)silane	C8	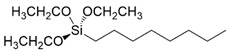
Hexadecyltrimethoxysilane	C16	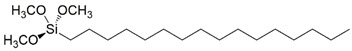
3,3,3-Trifluoropropyl-trimethoxysilane	F3	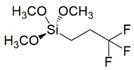

**Table 2:** Roughness values of Ra and comparison of the contact angles of Wenzel (θ_W_) and Young (θ_Y_) of the coatings.

Code	Ra [μm]	θ_W_ [°]	θ_Y_ [°/μm]
Comm.	0.73 ± 0.07	73.32 ± 1.77	66.84 ± 1.77
G/C2	0.85 ± 0.04	107.73 ± 1.74	110.99 ± 1.74
G/C8	0.96 ± 0.05	109.86 ± 1.57	110.72 ± 1.57
G/C16	0.53 ± 0.03	114.99 ± 1.20	142.85 ±1.20
G/F3	0.84 ± 0.08	107.67 ± 2.16	111.18 ± 2.16

**Table 3:** Mechanical characteristics of the coating.

Code	E [MPa]	σ_max_ [MPa]	σ_r_ [MPa]	ε_r_ %	load r [N]	W_r_ [J]
Comm.	29.71 ± 1.33	0.13 ± 0.02	0.0001 ± 0.0001	10.43 ± 0.93	0.01 ± 0.01	0.0004 ± 0.0002
G/C2	95.44 ± 0.62	0.54 ± 0.01	0.47 ± 0.0082	19.19 ± 0.32	36.02 ± 0.13	0.0017 ± 0.0001
G/C8	163.59 ± 1.63	0.94 ± 0.01	0.82 ± 0.0042	15.80 ± 0.28	62.77 ± 0.19	0.0069 ± 0.0001
G/C16	208.25 ± 0.43	1.64 ± 0.01	1.20 ± 0.0001	19.70 ± 0.21	92.23 ± 0.021	0.0219 ± 0.0002
G/F3	56.94 ± 0.91	1.05 ± 0.03	0.61 ± 0.0086	32.28 ± 0.41	46.34 ± 0.44	0.0372 ± 0.0002

## Data Availability

Not applicable.
